# The *Schizosaccharomyces pombe* Checkpoint Kinases Chk1 and Cds1 Are Important for Cell Survival in Response to Cisplatin

**DOI:** 10.1371/journal.pone.0006181

**Published:** 2009-07-09

**Authors:** Domenica Paparatto, Dane Fletcher, Karen Piwowar, Kimberly Baldino, Charlotte Morel, Stephen Dunaway

**Affiliations:** Department of Biology, Drew University, Madison, New Jersey, United States of America; Duke University Medical Centre, United States of America

## Abstract

**Background:**

DNA damage checkpoints insure that the integrity of genomic DNA is faithfully maintained throughout the eukaryotic cell cycle. In the presence of damaged DNA, checkpoints are triggered to delay cell cycle progression to allow for DNA repair. In fission yeast, the kinases Chk1 and Cds1 are major components of these DNA damage checkpoint pathways. Both Chk1 and Cds1 are important for viability in the presence of several DNA damaging agents. In this study we hypothesized that Chk1 and Cds1 play a vital role in fission yeast cells ability to survive exposure to the DNA damaging agent cisplatin. Cisplatin is a potent chemotherapeutic drug that interacts with DNA and causes both inter- and intra-strand DNA cross-links.

**Methodology/Principal Findings:**

Here, we demonstrated that treatment with cisplatin in fission yeast causes a Chk1-dependent DNA damage signal. *chk1* ^−^ cells were sensitive to cisplatin and Chk1 was phosphorylated in response to cisplatin treatment. We also showed that a Chk1-dependent DNA damage checkpoint pathway is activated in a dose-dependent fashion in cells challenged with cisplatin. Furthermore the Cds1 checkpoint kinase was also important for viability in cisplatin challenged cells. In *cds1* ^−^ cells, cisplatin treatment reduced cell viability and this phenotype was exacerbated in a *chk1* ^−^/*cds1* ^−^ background.

**Conclusions/Significance:**

Thus, we conclude that the concerted effort of both major checkpoint kinases in fission yeast, Chk1 and Cds1, protect cells from cisplatin induced DNA damage. These observations are significant because they suggest that various classes of inter-strand crosslinking agents may generate slightly different lesions as work by others did not observe loss of viability in *cds1* ^−^ cells treated with other crosslinking agents like nitrogen mustard.

## Introduction

Cell cycle checkpoints are signal transduction pathways that control the stepwise progression of cell division and arrest the cell cycle in the presence of damaged DNA, replication stresses or spindle disruption. As cells divide, their genome is under constant threat by intrinsic damage and exogenous agents that cause DNA lesions. Failure to repair these lesions can lead to mutation of the DNA, resulting in genomic instability, and cancer in higher eukaryotes. The DNA damage checkpoint specifically delays entry into mitosis in the presence of damaged DNA to provide cells enough time to repair damaged DNA before permanent mutations are made [1]. Many of the proteins involved in the checkpoint pathway have been identified genetically in budding and fission yeasts and are well conserved throughout eukaryotic evolution. Mutation or deletion of these proteins leads to a failure of cells to arrest cell cycle progression in the presence of DNA damage and manifests itself as a profound loss of cell viability [2]–[8].

In fission yeast, a major DNA damage induced cell cycle checkpoint occurs at the G2/M border and the protein kinase Chk1 is an essential component of this response. Deletion of *chk1^+^* results in both sensitivity to DNA damaging agents and checkpoint defects, such as a failure to restrain cell cycle in the presence of damaged DNA [4], [9], [10]. Chk1 homologs have been found in all organisms studied [11]. Chk1 kinase activity is activated by DNA damage and is dependent on several upstream “rad” components of the checkpoint signaling pathway (Rad1, Rad9, Hus1, Rad26, Rad3 and Crb2) [3], [6], [10], [12]–[15]. Overexpression of Chk1 can partially rescue the UV sensitivity of other ‘rad’ checkpoint mutants further implicating it as a downstream component of the checkpoint pathway [4].

In response to many types of DNA damage, Chk1 is phosphorylated on amino acid S345 and this event leads to the activation of the kinase activity of Chk1, which triggers a G2/M checkpoint arrest [9], [10], [12]. The DNA damaged induced phosphorylation of S345 on Chk1 is dependent on Rad3 in fission yeast. In mammals this phosphorylation dependent activation of Chk1 is controlled by the Rad3 ortholog ATR (ATM and Rad3 related) and has been shown to occur directly in response to DNA damage [16]–[18]. Disruption or mutation of any of the ATM family members in mammals (ATM or ATR) or fission yeast (Rad3) leads to severe defects in DNA damage checkpoint signaling [3], [19]–[21].

Although fission yeast Chk1 is phosphorylated and activated by multiple types of DNA damaging agents, Chk1 is only weakly activated during replication stress induced after treatment with the replication inhibiting drug hydroxyurea [12]. This observation distinguishes Chk1 from the another major effector kinase of the cell cycle checkpoint apparatus, Cds1 [22]. Cds1 (Checking DNA Synthesis) was originally identified as a suppressor of a mutant allele of DNA polymerase α and is thought to oversee the cell cycle checkpoint in response to replication stress [23]. Cds1 is homologous to Rad53 in budding yeast and Chk2 in mammals [24]–[26]. Deletion of *cds1^+^* results in sensitivity to DNA damaging agents and replication inhibitors [22]. Additionally, Cds1 kinase activity is stimulated by exposure to UV light and is required to delay S phase progression after UV irradiation and hydroxyurea treatment [22], [27]. These observations implicate Cds1 in both the DNA damage and replication checkpoints operating during S phase. Activation of Cds1 requires the upstream ‘rad’ checkpoint proteins but not Chk1 [22]. Chk1 is not activated in the presence of the replication checkpoint inducing drug hyrdoxyurea; however, in a *cds1Δ* background Chk1 becomes strongly phosphorylated in response to hydroxyurea treatment [22]. This observation suggests there is some functional overlap between the two checkpoint effector kinases. Moreover, cells deficient in either Cds1 or Chk1 are somewhat sensitive to DNA damage, however, a *cds1Δ/chk1Δ* double delete is as profoundly sensitive as a deletion of the “upstream” components of the checkpoint pathway, like Rad3 [22], [28], [29].

The DNA damage checkpoint works by controlling the Y15 phosphorylation of the cyclin-dependent kinase Cdc2 [30]. Chk1 is thought to control the G2/M checkpoint response by indirectly regulating Cdc2, the main controller of G2/M transition in fission yeast. Specifically, Chk1 phosphorylates Wee1 and Cdc25, both regulators of Cdc2, in response to DNA damage to promote checkpoint arrest [31]–[33]. These observations suggest there is a direct link between DNA damage detection and cell cycle control pathways [32]–[35].

The focus of this study was to investigate the sensitivity of fission yeast cells lacking Chk1, Cds1 or both to the DNA crosslinking drug cisplatin, a chemotherapeutic drug that generates both inter- and intra-strand DNA crosslinks [36]. Previous studies in fission yeast have examined the roles Chk1 and Cds1 play in maintaining viability in response to other inter-strand crosslinking agents (ICL) like nitrogen mustard and mitomycin C (MMC) [37], [38]. The direct involvement of human Chk1 and Chk2 have also been investigated in response to cisplatin and although these proteins are highly conserved, functional differences do exist [18], [39]. Therefore, the more information gained from the study of Chk1 and Cds1 in fission yeast could provide further insight into the functions of the mammalian homologs. Here we demonstrate that fission yeast Chk1 is important for cell survival in response to cisplatin treatment. Furthermore, we show that Chk1 is phosphorylated and a Chk1-dependent DNA damage checkpoint response is triggered by cisplatin. We also show that *cds1Δ* cells display a moderate sensitivity to cisplatin that is exacerbated in a *cds1Δ/chk1Δ* double delete. Our results suggest that in response to cisplatin, both the Chk1 and Cds1 pathways function together to maintain cell viability.

## Results

### *chk1Δ* strains are sensitive to cisplatin

**Figure 1:**
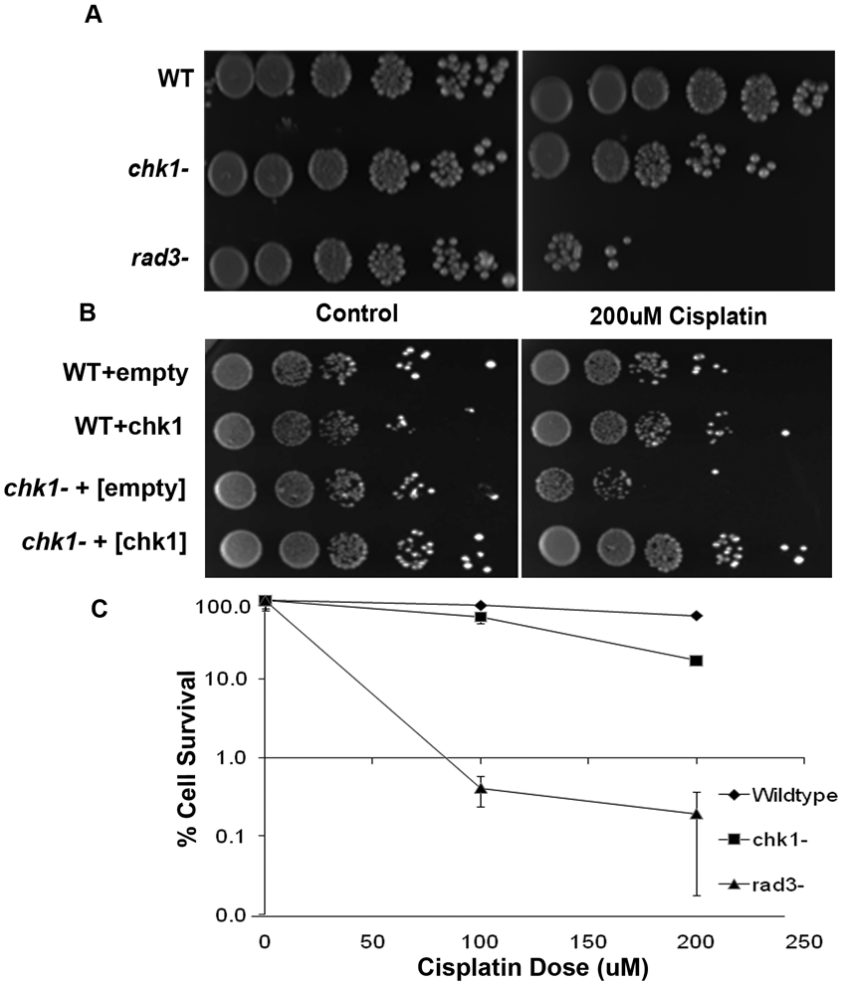
Deletion of *chk1^+^* results in cisplatin sensitivity. A. The wildtype, *chk1Δ*, and *rad3Δ* cells were grown to mid-log phase in YEA media, treated with 200 µM cisplatin for 1 hour, resuspended to 1×10^8^cells/ml and serially diluted and spotted on YEA plates. Plates were photographed after three days of growth at 30°C. B. The wildtype and *chk1Δ* cells transformed with an either empty pSP1 or pSP1-*chk1^+^* were grown to mid-log phase in SC-leu media, treated with 200 µM cisplatin for 1 hour, resuspended to 1×10^7^cells/ml and serially diluted and spotted on SC-leu plates. Plates were photographed after four days of growth at 30°C. C. The wildtype, *chk1Δ*, and *rad3Δ* cells were grown to mid-log phase in YEA media, treated with either 100 µM or 200 µM cisplatin for 1 hour and cells plated in triplicate on YEA and incubated for 3 days. Mean survival was calculated as a percentage of colonies appearing on untreated plates. Data represented here is typical of one of 3 independent experiments. Error bars represent standard deviation of the mean.

To further investigate the response of the fission yeast *Schizosaccharomyces pombe* to ICL agents, we determined the sensitivity of a strain lacking the DNA damage checkpoint kinase, Chk1, to cisplatin (Fig. 1a,c). Cells were grown to mid-log phase, treated with 200 µM cisplatin and 10 fold serial dilutions plated on YEA plates (Fig. 1a). When compared to wildtype cells, *chk1Δ* cells were sensitive to cisplatin and this sensitivity was intermediate when compared to *rad3Δ* cells (Fig. 1a). To ensure that these effects were due to the loss of *chk1^+^*, and not due to the acquisition of a suppressor we re-introduced the *chk1^+^* gene on a high copy plasmid. When transformed into the *chk1^−^* strain, the plasmid-borne copy of the *chk1^+^* gene was able to rescue the cisplatin sensitivity, whereas, the strain transformed with an empty plasmid remained sensitive to cisplatin treatment (Fig. 1b).

To confirm the observation made in Figure 1a that *chk1Δ* cells were sensitive to cisplatin, colony counting assays were performed in which cells were grown to mid-log phase, treated with either 100 µM or 200 µM cisplatin and 750 cells were plated on YEA. These plates were then incubated for 3 days at 30°C and scored for colony survival (Fig. 1c). *chk1Δ* cells displayed 62% survival when challenged with 100 µM cisplatin compared to 86% survival of a wildtype strain (Fig. 1c). As a control *rad3Δ* cells displayed less than 1% survival after 100 µM cisplatin treatment (Fig. 1c). At 200 µM cisplatin concentration viability decreased to 17% for *chk1Δ* cells and 64% for wildtype cells (Fig. 1c). Again, *rad3Δ* cells served as a control and displayed less than 1% survival after 200 µM cisplatin treatment (Fig. 1c). The results from these experiments revealed that *chk1Δ* cells display a dose-dependent sensitivity to cisplatin compared to wild type cells and this sensitivity was not as severe as the extreme sensitivity observed in *rad3Δ* cells (Fig. 1c).

Together, these results indicate that *chk1^+^* is important for the viability of fission yeast strains challenged with the DNA crosslinking agent cisplatin.

### Chk1 is phosphorylated and activates a DNA damage checkpoint arrest in response to cisplatin treatment

**Figure 2:**
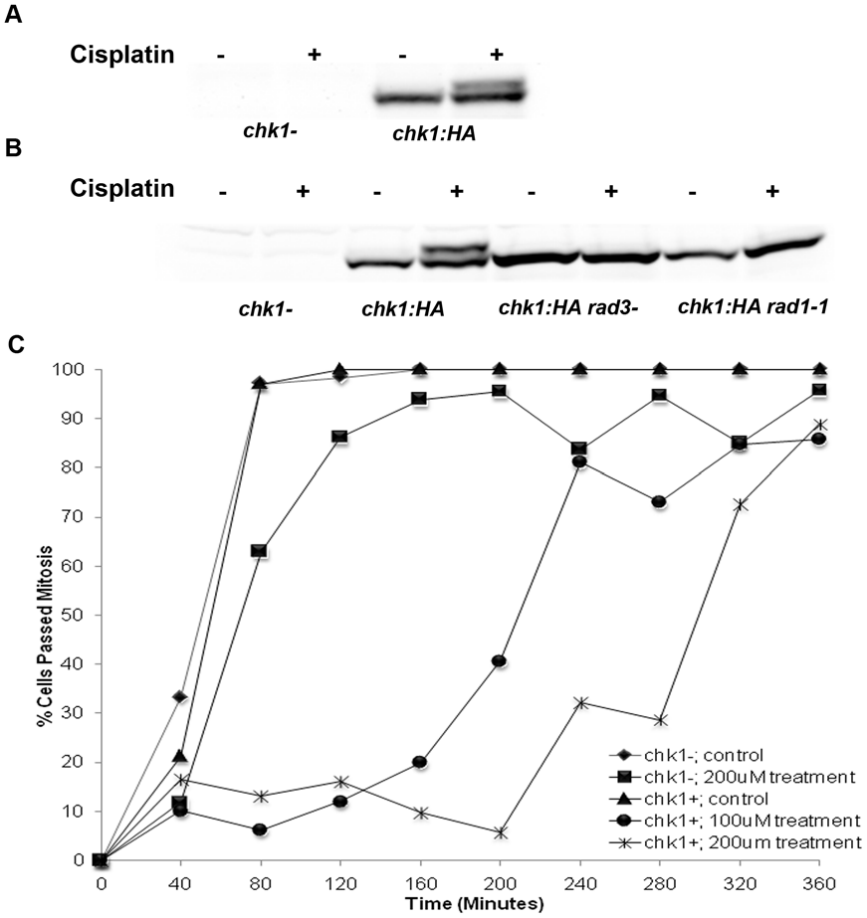
Cisplatin causes Chk1 to become phosporylated and activates a Chk1-dependent DNA damage checkpoint. A and B. Cultures from *chk1Δ*, *chk1-HA*, *rad3Δchk1-HA*, and *rad1-1chk1-HA* were grown to mid-log phase in YEA media at 30°C and treated with 200 µM cisplatin for one hour then harvested and analysed by western blot analysis with an antibody directed against the HA epitope tag at the C- terminus of Chk1. C. The chk1cdc25-22 and chk1Δcdc25-22 strains were grown to mid-log phase at 25°C and shifted to 36.5°C for 3 hours to synchronize cells in G2 phase of the cell cycle. This was followed by treatment with either 100 µM or 200 µM cisplatin for one hour at 36.5°C and then the strains were released to 25°C to permit cycling. The percentage of binulceate cells was scored over a 4 hour time period as an indication of cells progressing into and thru mitosis.

Given the observations that *chk1Δ* strains are sensitive to treatment with the DNA crosslinking agent cisplatin we investigated whether Chk1 was activated in cells treated with cisplatin. To address this issue, we performed western blot analysis on a fission yeast strain that expressed a functional hemmaglutinin tagged (HA) allele of *chk1^+^* (*chk1:HA*) in the presence or absence of cisplatin (Fig. 2a,b). The *chk1:HA* expressing strain and a *chk1Δ* strain were grown to mid-log phase and the indicated strains were treated with 200 µM cisplatin for one hour and subject to SDS-PAGE and western blot analysis with an antibody directed against the HA epitope tag. Whereas, the protein lysate from the untreated strain only contained a single species of Chk1 protein, the lysate from the cisplatin treated cells contained a second slower migrating form of Chk1 (Fig. 2a). It has previously been reported that Chk1 protein becomes phosphorylated in response to exposure to a variety of different DNA damaging agents like UV light and camptothecin, and this phosphorylation event results in the gel mobility shift of Chk1 [12], [40]. To confirm the identity of the bands observed in the western blots as Chk1, a *chk1Δ* strain was used as a negative control (Fig. 2a,b).

Next, we investigated whether the phosphorylation of Chk1 induced by cisplatin was dependent upon the upstream components of the DNA damage checkpoint response pathway. To accomplish this, we performed western blot analysis on protein lysates harvested from strains containing the HA-tagged allele of *chk1^+^* but lacking either *rad1^+^* or *rad3^+^* (*chk1:HA, chk1:HArad3Δ, chk1:HArad1-1*). Rad1 is a component of the Rad1, Rad9, Hus1 (9-1-1) complex of proteins which loads onto to chromatin and plays a role in phosphorylating Chk1 in response to DNA damage (as reviewed by [41]). Both *rad1^+^* and *rad3^+^* function upstream of *chk1^+^* in the checkpoint response pathway. When these strains were treated with cisplatin only the wild type strain expressing Chk1:HA contained phosphorylated Chk1. Chk1:HA in the *rad1-1* and the *rad3Δ* strains failed to phosphorylate Chk1 (Fig. 2b). Taken together we conclude that Chk1 becomes phosphorylated in response to cisplatin treatment and that this event is dependent on the upstream components of the DNA damage checkpoint response pathway in fission yeast.

We also investigated whether cisplatin activated a Chk1-dependent DNA damage checkpoint response. We used strains harboring the temperature sensitive allele *cdc25-22* either containing or lacking *chk1^+^*. At the restrictive temperature of 36.5°C cells expressing this allele arrest in G2, prior to mitosis, with an elongated phenotype. When returned to the permissive temperature of 25°C the cells can enter mitosis. These cells were arrested at G2 by shifting the culture to the restrictive temperature 36.5°C, treated with cisplatin, and released at the permissive temperature 25°C. Bi-nucleated cells were scored as an indication of cells that had entered or completed mitosis. Cells containing *chk1^+^* were able to delay mitotic entry in a dose-dependent fashion when challenged with cisplatin, whereas, *chk1Δ* cells were unable to do so and proceeded into mitosis (Fig. 2c). This premature entry into mitosis observed in the *chk1Δ* cells most likely results in the inability to faithfully repair cisplatin-induced DNA damage resulting in the loss of viability as observed in Figure 1a,b. Taken together this data supports the hypothesis that Chk1 is important for cell viability in the presence of cisplatin and that this cell viability is maintained by Chk1's ability to trigger a DNA damage checkpoint response in response to the cisplatin treatment. Furthermore, the duration of the cell cycle delay is dose dependent consistent with the notion that the delay allows time for repair of the cisplatin-induced damage.

### Chk1 and Cds1 act together to promote cell survival in response to cisplatin

**Figure 3:**
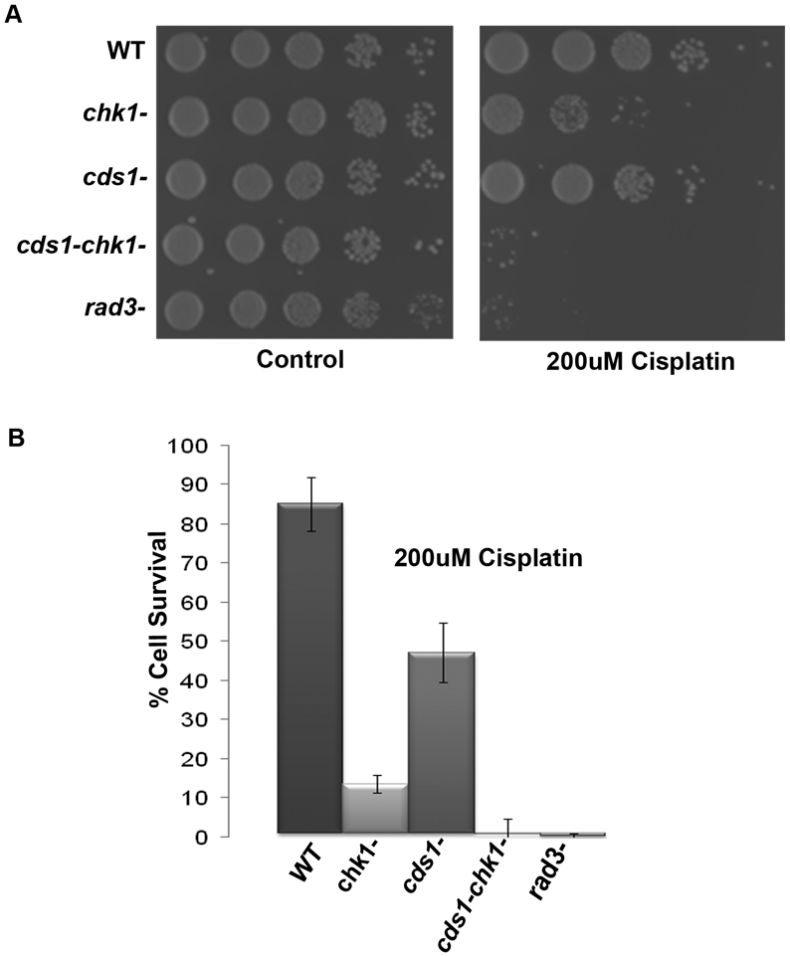
Both Chk1 and Cds1 are required to maintain cell viability in the presence of cisplatin induced damage. A. The wildtype, *chk1Δ*, *cds1Δ*, *chk1Δcds1Δ* and *rad3Δ* cells were grown to mid-log phase in YEA media, treated with 200 µM cisplatin for 1 hour, resuspended to 1×10^7^cells/ml and serially diluted and spotted on YEA plates. Plates were photographed after three days of growth at 30°C. B. The wildtype, *chk1Δ*, *cds1Δ*, *chk1ΔcdsΔ*, and *rad3Δ* cells were grown to mid-log phase in YEA media, treated with or 200 µM cisplatin for 1 hour and cells plated in triplicate on YEA and incubated for 3 days. Mean survival was calculated as a percentage of colonies appearing on untreated plates. Data represented here is typical of one of 2 independent experiments. Error bars represent standard deviation of the mean.

Although Chk1 mediates a major G2 checkpoint that delays mitosis, additional checkpoint pathways exist that delay entry into or progression through S phase. One of these pathways operates during the DNA synthesis phase of the cell cycle and this checkpoint is mediated by the Rad3-Cds1 pathway [11], [27]. We investigated whether Cds1 also played a role in maintaining cell viability in response to cisplatin as reports suggest that Chk1 and Cds1 may functionally overlap to protect cells against certain types of DNA damage [22]. We performed serial dilution spotting of cells that were treated with 200 µM cisplatin (Fig. 3a). *cds1Δ* cells were only slightly sensitive to cisplatin as compared to WT cells, whereas *chk1Δ* and *rad3Δ* cells displayed greater sensitivity. Strikingly, *chk1Δ/cds1Δ* double delete cells displayed an additive sensitivity to cisplatin treatment comparable to *rad3Δ* cells (Fig. 3a). This observation was confirmed by colony counting experiments where *cds1Δ* and *chk1Δ* cells displayed 47% and 14% survival when treated with 200 µM cisplatin. Whereas, the *chk1Δ/cds1Δ* delete cells displayed only 1% survival, similar to that observed in *rad3Δ* cells (Fig. 3b). We also observed a mild sensitivity of *cds1Δ* cells to nitrogen mustard in our colony counting assay (data not shown). Together these data suggest that both Chk1 and Cds1 checkpoint kinases appear to play a role in protecting cells from cisplatin and additively they cooperate to resolve both inter-and intra-strand crosslinking.

## Discussion

The results of our studies demonstrate that the DNA damage checkpoint effector kinase Chk1 plays a vital role in maintaining fission yeast cell viability in the presence of cisplatin induced DNA damage. Cells lacking Chk1 lose viability following cisplatin treatment (Fig. 1a,c). The cisplatin survival defect demonstrated here can be rescued by re-introduction of *chk1^+^* on a high copy plasmid further implicating Chk1 (Fig. 1b). This observation agrees with the importance of Chk1 in response to the inter-strand cross-linking agents nitrogen mustard and MMC [37]. The fission yeast Chk1 protein is also activated after cisplatin treatment as evidenced by the appearance of a phosphorylated form of Chk1 (Fig. 2a,b). These observations are consistent with those reported for human Chk1 as Zhao and Piwinica-Worms demonstrated that human Chk1 was phosporylated in response to cisplatin treatment [18]. Furthermore, this DNA damage mediated activation of Chk1 is dependent on the upstream ‘rad’ components of the DNA damage checkpoint response pathway as has been reported for other DNA damaging agents like UV, γ, and camptothecin (Fig. 2b) [12], [40].

Additionally, we demonstrated in this study that cisplatin triggered a Chk1-dependent DNA damage checkpoint arrest (Fig. 2c). The duration of the delay is extended with higher doses of cisplatin indicating that the DNA damage generated by cisplatin does indeed trigger a G2/M checkpoint response in *Schizosaccharomyces pombe* as has been demonstrated to occur in response to other crosslinking drugs like nitrogen mustards and MMC and in the budding yeast in response to cisplatin [37], [42]. In this study, we also investigated whether the replication checkpoint effector kinase Cds1 was involved in maintaining cell viability in response to cisplatin damage as has been reported for other DNA damaging agents, such as, UV, HU, and γ irradiation [22]. Specifically, our sensitivity data show that *cds1Δ* cells displayed a mild sensitivity as compared to wildtype cells and that *cds1Δ*/*chk1Δ* cells displayed an ‘additive’ loss of viability comparable to that observed in *rad3Δ* cells upon cisplatin treatment (Fig. 3a,b). The ‘additive’ phenotype observed in the *cds1Δ*/*chk1Δ* cells is in complete agreement with similar observations observed in the *cds1Δ*/*chk1Δ* double delete cells in response to UV and γ irradiation [22]. It has previously been reported in fission yeast that *cds1Δ* cells were not sensitive to other types of crosslinking agents, like nitrogen mustard, and in fact displayed a slight resistance to those drugs. However, *cds1Δ*/*chk1Δ* double delete cells displayed slightly more sensitivity than *chk1Δ* cells in those studies, consistent with our cisplatin results [37]. The difference in sensitivity of *cds1Δ* cells between these two studies with regards to cisplatin and other crosslinking drugs like nitrogen mustard is most likely explained by a difference in experimental procedure. When we performed our colony counting assay with nitrogen mustard we observed sensitivity in the Cds1 deficient strain similar to that observed in our cisplatin treated cells (data not shown). In the studies performed by Lambert et al, they observed that Cds1 kinase activity was upregulated in response to nitrogen mustard treatment to the same level observed with the common replication checkpoint inducing agent, HU [37]. This result suggested that Cds1 plays some role in how cells respond to crosslinked DNA which is consistent with our results. Additional evidence implicating Cds1 in this response pathway comes from studies of Cds1 orthologs in both budding yeast (Rad53) and mammalian cells (Chk2). *Rad53Δ* budding yeast are sensitive to cisplatin and in multiple mammalian cell types Chk2 is phosphorylated and activated in response to cisplatin treatment [39], [42]. A Chk2-dependent checkpoint arrest has recently been shown to be triggered by the nitrogen mustard class bi-functional alkylating agent bendamustine [43].

Together our data supports the notion that the replication and DNA damage checkpoint responses in fission yeast work together to handle potential threats to genomic stability. Currently, mammalian Chk1 and Chk2 are chemotherapeutic targets for kinase inhibitor drugs like UCN-01 aimed at disrupting these checkpoint responses in response to DNA damage [44]. The goal of these approaches by others remains to develop clinically successful combinatorial chemotherapies using drugs like UCN-01 and cisplatin in attempt to increase tumor cell killing, while at the same time reducing the requirement for high doses of chemotherapeutic drugs, like cisplatin that are known to be highly toxic to patients. Fission yeast has proven an extremely powerful tool in elucidating the DNA damage checkpoint pathways that have clinical relevance. We are currently attempting to develop cisplatin analogs to look at whether modifications of the lead compound alter the checkpoint response with the hope of further defining the cellular response pathway. The data from those studies could prove useful in the further design of kinase inhibiting drugs aimed at eliminating Chk1 or Cds1/Chk2 activity.

## Materials and Methods

### Yeast strains, media, and growth conditions

Yeast strains used in this study are listed in Table 1. Strains were grown in SC medium with appropriate amino acid supplements at 75 µg/ml or in rich yeast extract (YE) medium supplemented with adenine. Standard recipes were used for YE [45]. Strains were grown at 30°C unless otherwise indicated.

**Table 1:** Yeast Strains Used in This Study.

Strain	Genotype
972	*h^−^* (wild type)
NW158	*h^+^ chk1::ura4 ura4-D18 leu1-32 ade6-216*
NW240	*h^−^ rad3::LEU2 leu1-32 ura4-D18 his3*
KB1	*h^−^ leu1-32/pSP1* (empty plasmid)
KB2	*h^−^ leu1-32/pSP1-chk1:ep*
KB3	*h^+^ chk1::ura4 ura4-D18 leu1-32 ade6/pSP1* (empty plasmid)
KB4	*h^+^ chk1::ura4 ura4-D18 leu1-32 ade6/pSP1-ck1:ep*
NW223	*h^+^ chk1:ep ade6-216 leu1-32*
NW238	*h^+^ chk1:ep ade6-216 leu1-32 rad1-1*
NW246	*h^−^ chk1:ep ade6-216 leu1-32 rad3::LEU2*
NW404	*h^+^ cdc25-22 chk1::ura4 ura4-D18 leu1-32 ade6-210*
SP532	*h^+^ cdc25-22 ade6-210 leu1-32*
NW268	*h^−^ cds1::ura4 ura4-D18 leu1-32*
NW371	*h^+^ chk1::ura4 cds1::ura4 ura4-D18 leu1-32 ade6-216*

### Yeast transformation

Strains were grown in YEA media to a density between 5×10^6^–1×10^7^ cells/ml. The cells were pelleted and washed with 20 mls of sterile water. Cells were then pelleted and washed with 20 mls 0.1 M lithium acetate, pH 4.9. The cells were pelleted and resuspended with 0.1 M lithium acetate at a concentration of 1×10^9^ cells/ml and incubated at 30°C without shaking for 30 minutes. 100 µl of cells were then aliquoted to a tube containing 2–5 µg of plasmid DNA (pSP1-Chk1:HA), mixed gently and incubated at 30°C for 30 minutes. 290 µl of 50% polyethylene glycol-3350 was added, cells were gently mixed and incubated at 30°C for 30 minutes. Samples were then heat shocked at 42°C for 15 minutes. Cells were then pelleted and resuspended in 100 µl of SC-leu media and plated on proper selection plates.

### Cisplatin Survival Assay

Cisplatin (cis-diamineplatinumII dichloride) was obtained from Sigma (P-4394) and prepared in YEA media. Cells were grown to mid-log phase and treated with 100–200 µM cisplatin containing YEA media for one hour and then plated for 10-fold serial dilution spotting, individual colony counting, or used to assay DNA damage checkpoint activation as described [9].

### DNA damage checkpoint assay

To assay for the activation of the Chk1-dependent DNA damage checkpoint, a *cdc25-22* block release experiment was performed as previously described with minor modifications [46]. The *cdc25-22* strains used here were arrested at the restrictive temperature of 36.5°C for three hours followed by a one hour treatment with the indicated doses of cisplatin (Sigma P-4394) at 36.5°C.

### Lysate preparation and immunoblotting

Cells were harvested by centrifugation and lysed in phosphate buffered saline (PBS) containing Complete Protease Inhibitor (Roche Diagnostics) using glass beads and a FastPrep (Bio101) cell disruptor. Supernatant was collected from the lysed cells by centrifugation at 3000 rpm for 5 minutes. Aliquots were separated on Biorad 7.5% SDS-PAGE gels (161-1100), transferred to Biorad Immun-Blot PVDF membrane (162-0218), and probed with 12CA5 antibody to detect the HA epitope at the c-terminus of Chk1. Blocking of the membranes and all antibody incubations and washes were performed in 1% milk and 0.05% Tween-20 in PBS. 12CA5 antibody was used at 1∶500 and a peroxidase coupled secondary antibody (Santa Cruz-sc-2005) was used at 1∶1000. The SuperSignal West Dura Extended Duration Substrate (Thermo Scientific-34075) was used for detection. Membranes were then detected in an Alpha Innotech FluorChem SP imaging system.
